# Progress and perspectives of platinum nanozyme in cancer therapy

**DOI:** 10.3389/fchem.2022.1092747

**Published:** 2022-12-02

**Authors:** Xi Wang, Xueting He, Chaofan Liu, Weiheng Zhao, Xianglin Yuan, Rui Li

**Affiliations:** ^1^ Department of Oncology, Tongji Hospital, Tongji Medical College, Huazhong University of Science and Technology, Wuhan, China; ^2^ Department of Obstetrics and Gynecology, Graduate School of Medicine, The University of Tokyo, Tokyo, Japan

**Keywords:** platinum, nanozyme, tumor microenvironment, cancer therapy, clinical diagnosis

## Abstract

Malignant tumors, one of the worst-case scenarios within human health problems, are now posing an increasing threat to the well-being of the global population. At present, the treatment of malignant tumors mainly includes surgery, radiotherapy, chemotherapy, immunotherapy, etc. Radiotherapy and chemotherapy are often applied to inoperable tumors, and some other tumors after surgery as important adjuvant therapies. Nonetheless, both radiotherapy and chemotherapy have a series of side effects, such as radiation-induced lung injury, and chemotherapy-induced bone marrow suppression. In addition, the positioning accuracy of radiotherapy and chemotherapy is not assured and satisfactory, and the possibility of tumor cells not being sensitive to radiation and chemotherapy drugs is also problematic. Nanozymes are nanomaterials that display natural enzyme activities, and their applications to tumor therapy have made great progress recently. The most studied one, platinum nanozyme, has been shown to possess a significant correlation with radiotherapy sensitization of tumors as well as photodynamic therapy. However, there are still several issues that limited the usage of platinum-based nanozymes *in vivo*. In this review, we briefly summarize the representative studies regarding platinum nanozymes, and especially emphasize on the current challenges and the directions of future development for platinum nanozymes therapy.

## Introduction

Artificial nano biomimetic enzyme (nanozyme) has become increasingly prominent as a new option for adjuvant therapy (Jiang et al., 2019). Nanozyme is a kind of nanomaterial with natural enzyme activity (Golchin et al., 2017). It can imitate natural enzyme to realize catalytic reaction *in vivo* to improve tumor microenvironment or solve the problem of hypoxia around tumor by increasing oxygen content (Golchin et al., 2017). The cost of natural enzyme production is high, and its yield is low in content and quality. In contrast, nanozyme has relatively stable physical and chemical properties, easy preservation and low cost of production (Sutrisno et al., 2020). Nanozymes can catalyze chemical reactions under physiological mild conditions, and also possess some special physical and chemical properties (Huang et al., 2019), so it is widely used in the medical field.

Platinum nanozyme is one of the most widely studied nanozymes. It has multifunctional enzyme like catalytic properties. Platinum nanoparticles can catalyze the decomposition of hydrogen peroxide to generate oxygen to alleviate the hypoxia state in the tumor microenvironment, and this decomposition process is high in stability, efficiency and durability. Reactive oxygen species (ROS) produced by hydrogen peroxide catalyzed by platinum nanozymes can trigger immunogenic cell death (Zhu et al., 2022a). In addition, platinum nanozymes have been proved to be significantly related to radio-chemotherapy and photodynamic therapy for tumors. However, the treatment *in vivo* is still limited by some problems, such as lack of tumor specificity. In this review, we briefly summarized the representative studies of platinum nanozymes, and especially emphasized the challenges within this special therapy and its future development direction.

## Tumor microenvironment

In the process of its own development, the tumor created the specific microenvironment to meet its own growth needs (Xiang et al., 2019). This microenvironment is characterized by low oxygen content, acidic and high hydrogen peroxide level, and it will lead to the abnormal secretion of some cell growth factors, such as CAFs, PDGF and TGF-β (Semenza, 2012). These cytokines can protect tumor growth, facilitate tumor metastasis, invasion and adhesion (Graham and Unger, 2018), and at the same time, endanger the survival of surrounding normal tissue cells. This specific tumor microenvironment has seriously affected the therapeutic efficiency and efficacy of tumor related therapy.

### Hypoxia

Hypoxia is a major feature of tumor microenvironment, as tumor cells consume a large amount of oxygen in the process of rapid proliferation. The development of tumor is usually accompanied by the malformation of tumor blood vessels, which will also lead to hypoxia in the tumor microenvironment (Brown and Wilson, 2004). Hypoxia state will cause acidosis through the generation of some auxin and growth factors, and it has adverse effects on normal surrounding tissues and cells. However, hypoxia and abnormal blood supply play a certain role in promoting tumor growth, promoting tumor invasion and metastasis (Rankin and Giaccia, 2016). In addition, hypoxia in the tumor microenvironment will greatly reduce the efficacy of some treatment methods that require oxygen consumption, such as radiotherapy and photodynamic therapy (Yang et al., 2020). Therefore, adjusting the hypoxia state in tumor microenvironment will contribute to tumor therapy. At present, there are several methods to improve the oxygen in tumor microenvironment include hyperbaric oxygen therapy (Fernandez et al., 2021), HIF-1α pathway inhibitor (Maftouh et al., 2014) and inducing tumor vessel normalization (Pietrobon and Marincola, 2021). However, these traditional therapies also have adverse side effects and controversy. Recently, the role of nanomaterials in improving tumor microenvironment has been gradually revealed. Some nanomaterials can directly improve the oxygen by targeting tumor sites and delivering nanomaterials containing oxygen agents (Cheng et al., 2015; Yuan et al., 2021). In addition, the catalytic efficiency of nanozyme is much higher than other catalysts (Jia et al., 2016), and nanozyme has stable physical and chemical activity compared with natural enzyme, so it has strong adaptability to the harsh tumor microenvironment. Its production cost is also far lower than that of natural enzyme. Therefore, artificial nanozyme has significant advantages in the treatment for hypoxia state within tumors (Wu et al., 2019).

### High H_2_O_2_ content

Hydrogen peroxide is an intermediate product of cell metabolism (Yang et al., 2021). Under low concentration, hydrogen peroxide is a regulator of cell proliferation. Under high concentration, hydrogen peroxide will cause oxidative damage to DNA, leading to cell cycle arrest and eventually cell death. Compared with normal cells, tumor cells are more sensitive to the change of hydrogen peroxide level. It can even affect the malignant phenotype of tumor cells. It has been reported that hydrogen peroxide has dual effects in cancers-promoting uncontrolled cell growth or causing apoptosis in tumor cells. Hydrogen peroxide promotes cells proliferation mainly by consuming glutathione and increasing the expression of downstream protein kinase, thereby activating redox sensitive transcription factors and downstream genes. In addition, hydrogen peroxide can also promote the invasion of tumor cells by activating HIF-1, FAK/Src and other signal transduction pathways (Califano and Alvarez, 2017). However, excessive hydrogen peroxide will block cell cycle and cause cell apoptosis. In other words, too low or too high hydrogen peroxide content is intolerable to tumor cells.

**FIGURE 1:**
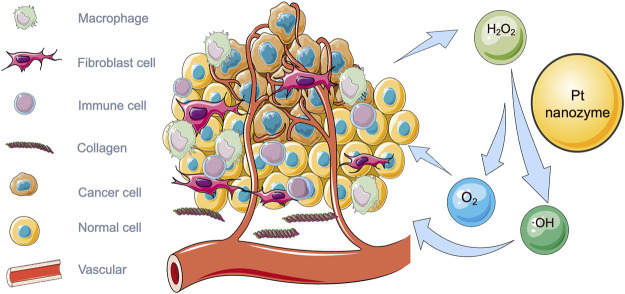
Illustration of tumor microenvironment and the catalytic effect of platinum nanozymes on hydrogen peroxide in tumor microenvironment.

Because of the low level of hydrogen peroxide in the microenvironment and the high expression of glutathione inside tumor cells, the production of ·OH can be promoted by increasing peroxidase-like activity. There is an increasing number of studies on nanozymes’ role in promoting catalase activity (Table 1), among which platinum nanozymes are capable of promoting the decomposition of endogenous hydrogen peroxide (Figure 1). Xu et al. (2022a) prepared a TME activated atomic engineering PtN_4_C monatomic nanozyme. The “butterfly effect” of ROS is induced by promoting intracellular hydrogen peroxide cycle and glutathione deprivation, as well as X-ray deposition of chemodynamic therapy (CDT) and O_2_-dependent chemoradiotherapy involving ROS. Through superior superoxide dismutase like activity, O_2_ is converted into hydrogen peroxide so as to continuously supplement endogenous hydrogen peroxide, alleviating the problem of insufficient hydrogen peroxide content in tumor microenvironment. Oxygen in tumor microenvironment can be produced by a self-circulating valence changed between Pt (IV) and Pt (II), which is how PtN_4_C-SAzyme increases the oxygen in tumor microenvironment.

**TABLE 1:** Pt nanozymes classification and application.

Nanozymes	Morphology	Particle size	Application	References
Hollow PtCo alloy nanospheres	Nanosphere	70 nm	Radiotherapy	Li et al. (2021a)
BiPt-PFA	Heterojunction structure	170 nm	Radiotherapy	Zhang et al. (2020)
PtN_4_C-SAzyme	Spheroid	110 nm	CDT, chemoradiotherapy	Xu et al. (2022a)
Pt-Carbon nanozyme	Mesoporous	122 nm	PDT	Yang et al. (2020)
CPT-TK-HPPH/PtNP	Spheroid	179 nm	PDT	Hao et al. (2020)
ICG-PtMGs@HGd	Octahedron	203.5 nm	PDT, PTT	You et al. (2020)
Fe_3_O_4_@Carbon@Pt-Chlorin e6 nanozyme	Yolk-shell	75 nm	PDT, PTT	Xu et al. (2022b)
Pt-CuS Janus	Hollow	285 nm	SDT, PDT	Liang et al. (2019)
Nano-Pt/VP@MLipo	Spheroid	120 nm	PDT	Liu et al. (2021)
CoO@AuPt nanozyme	Hollow	36 nm	CDT	Melamed et al. (2015)
CeO_2_/Au@Pt-PEG	Nanosphere	100 nm	PTT	Tang et al. (2022)
Pt-dendrimer nanozyme	Dendritic	<10 nm	Metastasis inhibition	An et al. (2021)
SFX/GOx/HGN@Pt	Spheroid	56 nm	Starvation therapy	Wu et al. (2021)
Ti_3_C_2_Tx-Pt-PEG	3D ball-and-stick model	200 nm	Starvation therapy	Zhu et al. (2022d)

Using the synergistic effect between iron atoms and Pt clusters, Chen et al. (2021) loaded Pt clusters on Fe-N nanozymes to synthesize highly active peroxidase-like nanozymes Fe_SA_-Pt_C_ nanozymes. The activity of the original nanozyme catalyzing hydrogen peroxide can be enhanced by 4.5 times (Chen et al., 2021).

## Platinum nanozyme for tumor treatment

### Radiotherapy

Radiotherapy occupies a very important position among current cancer treatment methods (Zhang et al., 2019), and more than half of tumor patients need radiotherapy during their whole treatment plans. Radiation therapy uses X-ray, high-energy electron beam and other rays to irradiate tumor tissues. The energy generated by a large number of rays can damage the chromosomes of cells, prevent cell growth and even cause cell death. The current mainstream radiotherapy technologies mainly adopt three-dimensional conformal radiotherapy, and meanwhile four-dimensional computed tomography (CT) brings patient-specific tumor motion into the measurement range (Vinod and Hau, 2020). Radiotherapy is the primary treatment for advanced cancer, and it is also applied to some patients without surgical treatment or after tumor operation (Ma et al., 2017). However, radiotherapy also has a variety of side effects. These are mainly due to the fact that radiation affects not only the tumor cells but also the surrounding normal tissues. In addition, there are also some other problems such as insufficient radiation deposition in the tumor tissue, aggravating the hypoxia of the tumor microenvironment, etc. In the meantime, vascular abnormalities in the tumor microenvironment will also contribute to hypoxia, and solid tumor tissues lead to radiation resistance (Dou et al., 2018). Hypoxic cells are more resistant to radiation than normal cells. This requires a higher dose of radiotherapy so as to kill tumor cells (Huo et al., 2017). But with the increasing dose of radiation, the damage from it to the surrounding normal tissue and cells will also elevate (Wolfson et al., 2011). Consequently, side effects of radiotherapy are usually related to the treatment doses since higher radiation will increase the toxicity to normal tissue. The bystander effect is one of them, and it refers to the response of the signal generated by the irradiated cells to the adjacent non-irradiated cells during irradiation (Lauber et al., 2012; Deloch et al., 2016).

In response to these side effects of radiotherapy, the new Pt nanomaterials are capable of improving the sensitivity towards radiotherapy, protecting the normal tissues around the tumor, and even reducing the side effects of radiotherapy.

**FIGURE 2:**
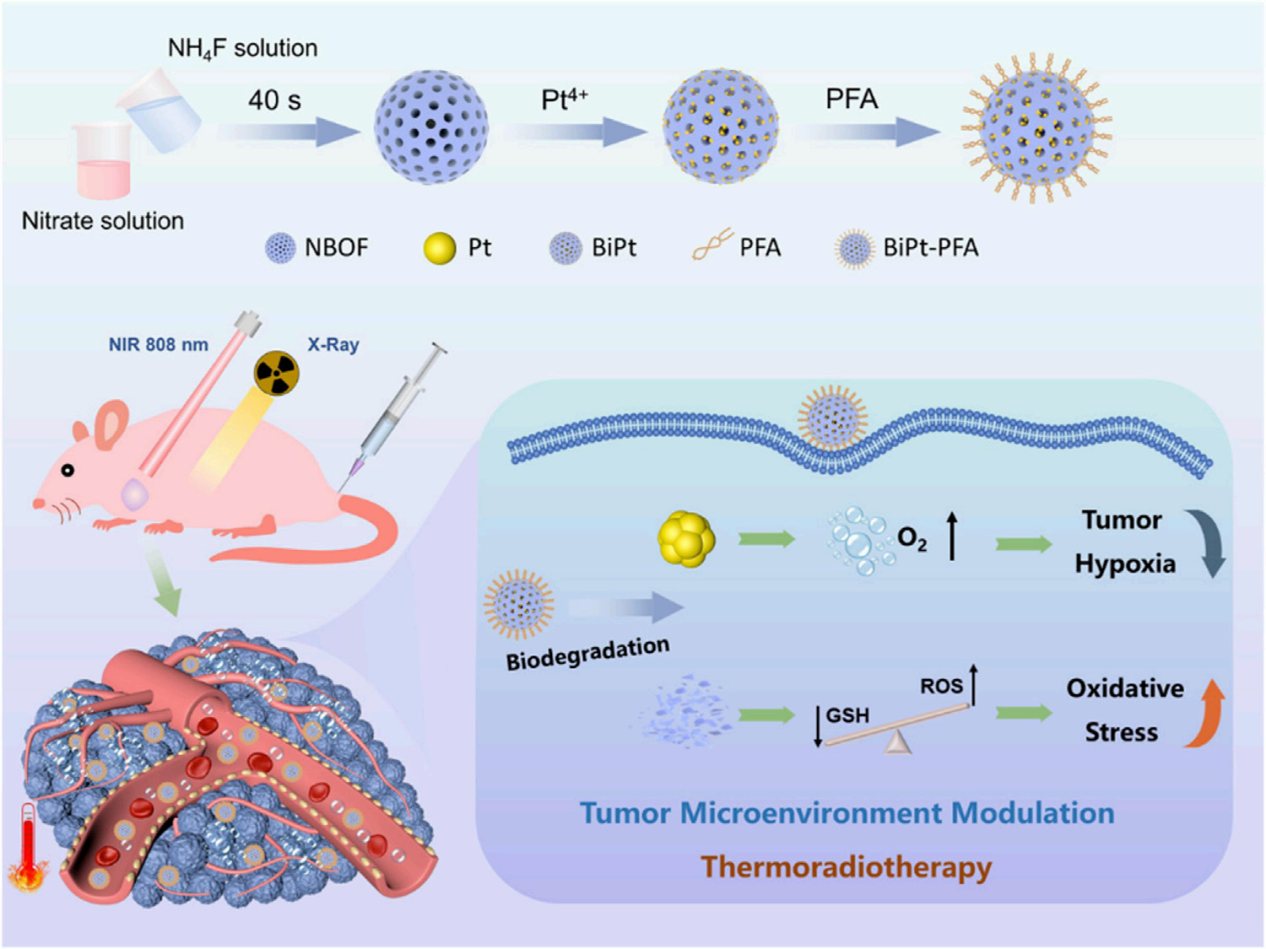
Schematic illustration of the synthesis of BiPt-PFA NPs and their application for synergistic thermoradiotherapy with hypoxic tumor (Zhang et al., 2020). Copyright ^©^ 2020 American chemical society.

Pt nanomaterials can promote endogenous hydrogen peroxide decomposition to produce more oxygen. Li et al. (2021a) proposed a PtCo nanosphere radiosensitizer, which can use the hollow structure to expand the specific surface area and further amplify the catalytic effect of Pt on hydrogen peroxide (Figure 2). The effect of this nanozyme material has been verified in both *in vivo* and *in vitro* experiments. This hollow structure of PtCo alloy nanosphere can significantly inhibits the tumor growth of non-small cell lung cancer.

The biodegradable composite nanomaterial BiPt-PFA produced by Zhang et al. (2020) can also be used as a radiosensitizer. They incorporated Pt NPs into mesoporous Na_0.2_Bi_0.8_O_0.35_F_1.91_: Yb (NBOF) to form BiPt heterostructures, and modified the surface with Polyethylene glycol (PEG)-modified FA to form BiPt-PFA, which increases the water solubility and targeting of this material. At the same time, this composite nanozyme material has a strong ray absorption capacity. The tumor-targeted BiPt-PFA absorbs radiation and decomposes hydrogen peroxide to produce oxygen, which alleviates the hypoxia of tumor microenvironment, increases ROS, and kills tumor cells. Further, the BiPt-PFA enriched at the tumor site can cooperate with the GSH to degrade NBOF and consume the intracellular GSH. Taken together, BiPt-PFA can modulate the tumor microenvironment and improve the anti-tumor effect of thermoradiotherapy.

Single-atom nanozyme (SAzyme) usually hold great promise in the field of cancer therapy (Zhu et al., 2022b). The single-atom platinum nanozyme with admirable superoxide dismutase-like and peroxidase-like activities studied by Xu et al. (2022a) could catalyze the conversion of superoxide anions (O_2_^•−^) into hydrogen peroxide, thus increasing the hydrogen peroxide level at the tumor site. Then, hydroxyl radicals (∙OH) and oxygen are generated which can reinforce CDT and chemoradiotherapy. Further, this single-atom platinum nanozyme showed better enzyme activity under the condition of low pH value, which is consistent with the acidity of the tumor microenvironment, thus leading to a better anti-tumor effect. More importantly, changes in the self-cycling valence of Pt (IV) and Pt (II) lead to sustained depletion of GSH and the release of large amounts of Pt^2+^, ultimately inhibiting antioxidant defense, and overcoming resistance. Altogether, this PtN_4_C-SAzyme has a significant effect on promoting CDT and chemoradiation sensitization.

### Photodynamic therapy

Photodynamic therapy (PDT), the treatment through intravenous injection of photosensitizers (Dougherty et al., 1998), is able to sensitize tumor tissue to light, catalyze substrate oxygen to produce reactive oxygen free radical ROS (Hao et al., 2020), and cause irreversible damage to tumor cells. In recent years, numerous studies have shown that PDT is one of the most effective minimally invasive cancer treatments which can eliminate malignancies without damaging the healthy tissue surrounding the tumor. Photosensitizers usually stay in tumor cells longer than normal cells, so that the amount of photosensitizer in tumor cells is higher than that in normal cells. When the photosensitizer is exposed to light irradiation, it will change from the ground state to the single point excited state, and then to the triple excited state. The excited photosensitizer will react with the surrounding medium to produce ROS to kill tumor cells. Compared with the traditional cancer treatments, PDT is much safer for patients. It has low toxicity, less damage to normal tissues, and relatively higher selectivity and tissue specificity to tumor cells. In addition, PDT also does not produce long-term immunosuppression and lacks treatment-related resistance mechanisms. Therefore, multiple repeated treatments can be performed. At present, PDT has been put to use clinically widely, such as Barrett’s esophagus, psoriasis, etc. (Zhang and Wu, 2018).

The effect of PDT is closely related to photosensitizers and requires sufficient light and oxygen to maintain the photochemical reaction steps (Sutrisno et al., 2020), but the oxygen concentration around the tumor is often very low, so this is one of the main problems of PDT. The rapid proliferation of tumor cells and abnormal increase of blood vessels lead to hypoxia state within tumor microenvironment, which will affect the production of ROS, as well as photochemical reactions. Thus, improving the hypoxia state in tumor microenvironment can greatly enhance the therapeutic efficiency of PDT.

Platinum nanozymes can improve the efficacy of PDT through a variety of ways, one of which is to enhance the efficacy of PDT by enhancing the catalytic activity of endogenous peroxidase to promote oxygen production. The platinum carbon integrated nanozyme constructed by Yang et al. (2020) uses platinum nanozyme to immobilize MOF-derived carbon nanozyme, thereby enhancing the catalytic performance of carbon nanozyme. It also enhances the intrinsic photothermal performance, and further improves the ability to kill tumor cells.

Cao et al. (2021) proposed to use polydopamine nanoparticles to stabilize the Pt catalyst, and turn into a nanoplatform for carrying photosensitizers and Pt NPs. Pt NPs can promote the catalytic of hydrogen peroxide in tumors, and generate oxygen to improve the effect of PDT on tumors. The cells cytotoxicity test was performed with human breast cancer cells co-cultured with folate labelled Pt-ICG@PDA, ICG@PDA and PDA nanoparticles. The experimental results showed that under the dark conditions, there was no significant difference among different groups, which proved that the PDA-based nanoparticles had good biocompatibility. After irradiation, the cells cultured with Pt-ICG@PDA showed a worse survival rate, indicating that Pt-ICG@PDA had a significant anti-tumor effect.

**FIGURE 3:**
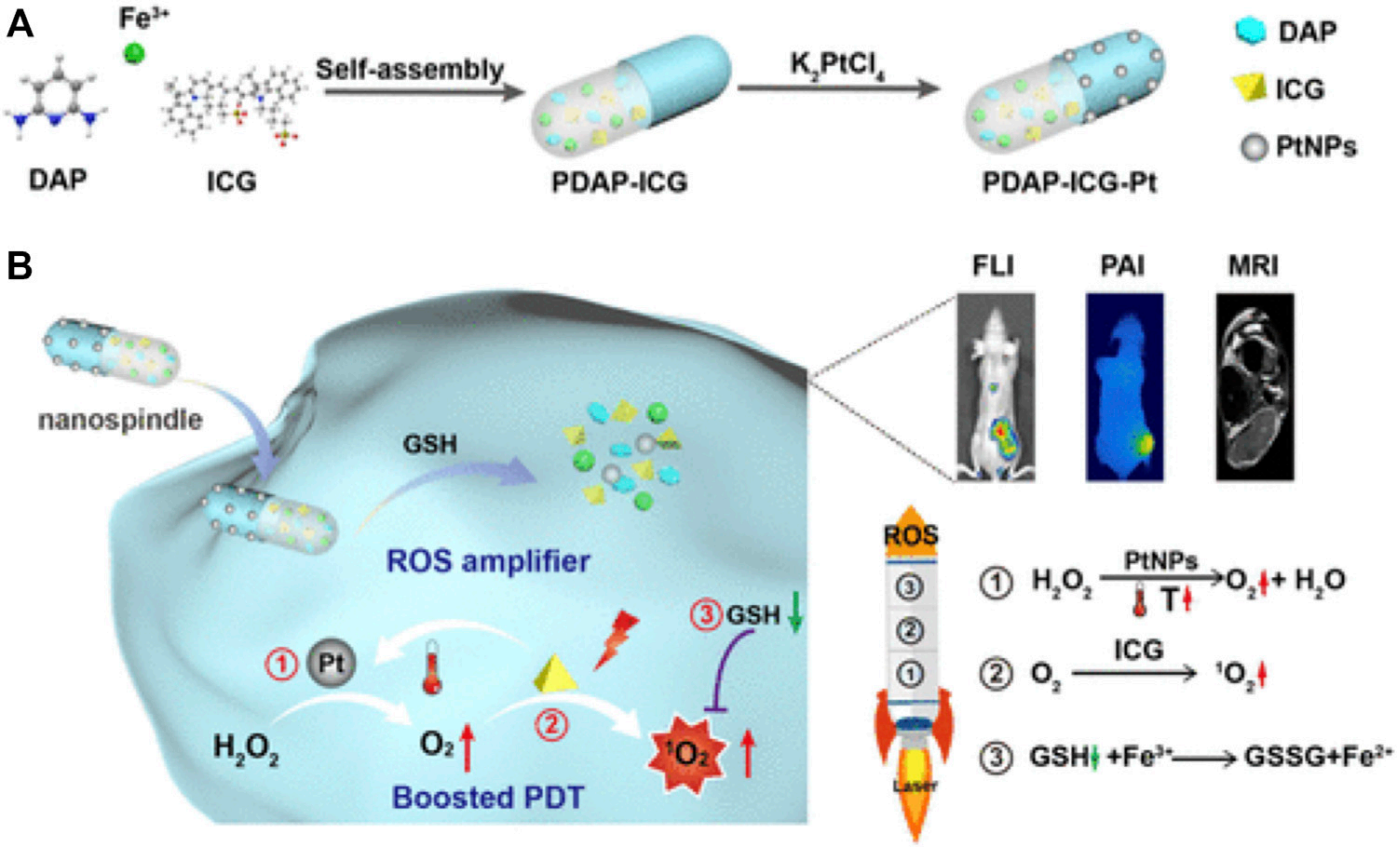
Illustration of the cascade strategy for enhanced PDT using rationally designed Nanofusiforms as a ROS amplifier; **(A)** synthesis of PDAP–ICG–Pt Nanofusiforms; **(B)** modulating the tumor microenvironment for enhanced PDT efficacy *via* nanoyzme-catalyzed and heat-improved oxygen self-supply to keep sustainable high ROS levels and antioxidant depletion to prevent PDT tolerance (Dong et al., 2021). Copyright ^©^ 2021 American chemical society.

PDAP-ICG-Pt NPs conducted by Dong et al. (2021) are a self-assembled fusiform nanozyme material, which can improve the photostability of indocyanine green (ICG) and endow Pt nanozymes with better stability and catalase activity. The continuously O_2_ self-supply and GSH depletion achieve cascade amplification to enhance the therapeutic effect of PDT (Figure 3).

The platinum nanozyme *in situ* mesoporous polydopamine constructed by Hu et al. (2019) also increases photodynamic therapy efficacy by improving the catalytic efficiency and generating more oxygen. And the effect of this nanozyme on inhibiting tumor growth has been verified by *in vivo* tests.

In addition to increasing the activity of catalase, the therapeutic effect of PDT can also be improved by increasing the scavenging activity of ROS. The CPT-TK-HPPH/Pt NP nanoparticles developed by Hao et al. (2020) have excellent ROS-related drug release behavior and enhanced PDT efficiency.

Metal-organic framework (MOF) is a porous structure composed of metal ions/clusters and organic ligands. Due to its porous structure, it is widely used in the field of drug delivery (Wang et al., 2018). In addition, the porous structure also provides a large specific surface area. When combined with Pt nanozyme, it can improve the rate of hydrogen peroxide decomposition of Pt nanozyme, accelerate the generation of oxygen, and alleviate the hypoxia state of the tumor microenvironment. The ICG-PtMGs@HGd NPs constructed by You et al. (2020) are Pt-decorated MOF@GNSs (PtMGs) NPs that are surface-modified with human serum albumin-chelated gadolinium (HSA-Gd, HGd) and loaded with ICG (ICG-PtMGs@HGd) to achieve synergistic PDT/PTT effect. The hollow structure is not only available in MOF, but also in the Prussian blue nanoparticles (HPBs) obtained by hydrothermal and hydrochloric acid etching of Prussian blue (PBs). Au-Pt@HPBs (APHPBs) is prepared by reduction method, that is Au-Pt nanozymes were grown *in situ* on the HPBs (Shen et al., 2022). APHPBs is a hollow structure, which is convenient for loading photosensitizer Ce6, so that the obtained Ce6-Au-Pt@HPB can effectively use Pt nanozyme to promote the decomposition of hydrogen peroxide, generate oxygen, convert it into ROS, and target and kill the tumor cells under 660 nm laser. The hollow structure of APHPBs can also expand the surface area of the reaction system. It is confirmed that this nanozyme not only effectively improved the efficacy of PDT, but also had biological safety *in vivo* experiments.

Compared with a single nanozyme, the composite nanozyme has fewer shortcomings. The yolk-shell structure in Fe_3_O_4_@Carbon@platinum-Chlorin e6 (MCPtCe6) can make Fe_3_O_4_ dispersed in the carbon shell and the carbon shell can be used as the carrier of Pt NPs. MCPtCe6 plays a role in the acidic and high concentrations of hydrogen peroxide tumor microenvironment through enzyme-like catalysis, which can enhance the decomposition ability of Pt nanozymes and significantly promote the treatment of PDT and PTT (Xu et al. 2022b).

Platinum nanozymes can also release platinum ions to kill tumor cells, but they have limited therapeutic effect on deep solid tumors because of the poor penetrability to tumor tissues. And platinum nanozymes have certain cytotoxicity to cells. Nano-Pt/VP@MLipo designed by Liu et al. (2021) encapsulate ultra-small nanozymes into liposomes by reverse evaporation technology. The photosensitizer verteporfin (VP) is loaded in the lipid bilayer and hybridized with the cell membrane of macrophages to obtain biomimetic properties and targeting. When nano-Pt/VP@MLipo reached the tumor site, oxygen generation catalyzed by nano-Pt boosted the VP-mediated PDT, which in turn triggered the release of nano-Pt through the membrane permeability. The ultra-small Pt nanozyme can penetrate the tumor better, while the oxygen produced also contributes to the penetration of the tumor, thereby enhancing the chemotherapy effect. Moreover, no obvious side effects were found in animal models.

As mentioned above, Pt nanozymes’ role in promoting PDT has been fully testified in these researches.

### Sonodynamic therapy

Sonodynamic therapy (SDT) is also a new non-invasive cancer treatment. The difference between sonodynamic therapy and photodynamic therapy is that SDT can activate ultrasound sensitizers, promote the generation of ROS, thereby killing tumor cells. Compared with photodynamic therapy, it has stronger penetration power, and thus can achieve good therapeutic effects on deep solid tumor cells (Pan et al., 2018). SDT has significant advantages in tumor treatment, yet so far, almost all of the ultrasound sensitizers that have been studied are flawed, such as poor chemical stability. Hypoxia in the tumor microenvironment remains a key factor limiting the therapeutic effect of SDT.

**FIGURE 4:**
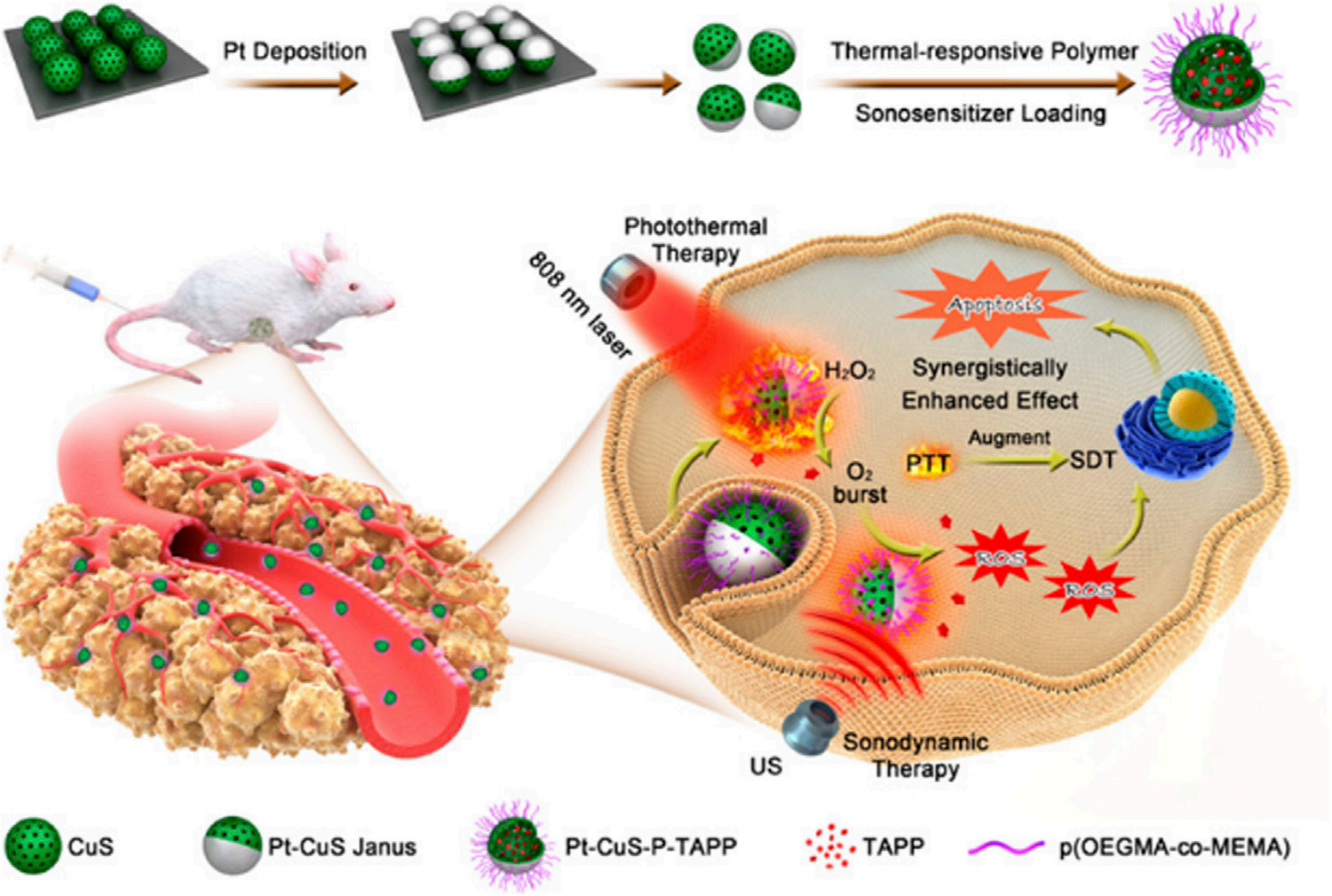
Schematic illustration of the main synthesis procedures and antitumor mechanism of PCPT (Liang et al., 2019). Copyright ^©^ 2019 American chemical society.

In the study reported by Liang et al. (2019), they synthesized a novel Pt-CuS Janus NP which is composed of hollow CuS and Pt. The hollow CuS structure has a large internal cavity and can be used to load sonosensitizer molecules to achieve SDT. Compared with CuS NPs, Pt deposition not only improves photothermal properties due to enhanced local electric field, but also has nanozyme activity to catalyze endogenous overexpressed H_2_O_2_ to produce O_2_, thereby overcoming hypoxia and increasing SDT-induced production of highly toxic ROS. Moreover, the heat produced by Pt-CuS during 808 nm laser irradiation can enhance the catalytic activity of Pt and increase the O_2_ level, further promoting the effect of SDT. This synergistic catalytically augmented SDT efficiency and highly photothermal effect has been validated *in vivo* and *in vitro* experiments, along with a high biological safety of the treatment (Figure 4).

In addition, the therapeutic effect of SDT can also be improved by preparing the MOF based on Pt nanozyme (DOX@PCN-224/Pt). Due to the large surface area, porous structure, excellent drug delivery and release ability of MOF, DOX@PCN-224/Pt can potently promote the rapid diffusion of O_2_ and ^1^O_2_, deregulating the expression of hypoxia-inducible factor *α*, thus improving the effect of SDT and chemotherapy (Ren et al., 2022).

### Chemotherapy

Chemotherapy remains one of the most popular strategies for malignant tumors. However, they usually lack site targeting and sustained release, and the accompanying drug resistance and systemic toxic enrichment limit their application.

The liposome of platinum nanozyme studied by Liu et al., (2021) can promote the penetration of ultra-small platinum nanozyme into deep tumor tissue, and the oxygen generated by catalysis can enhance the chemotherapy effect of verteporfin.

### Chemodynamic therapy

Chemodynamic therapy (CDT) is a novel tumor treatment technology first proposed in 2016. It is based on the transformation reaction of endogenous chemical reaction products of tumors (Xu et al., 2022a). In 1893, chemist Fenton HJ found that the mixed solution of hydrogen peroxide and ferrous ion had strong oxidation. This chemical reaction was later confirmed:

$${Fe}^{2+}+H_{2}O_{2}\to {Fe}^{3+}+{OH}^{-}+\cdot OH$$

The hydroxyl radical in the product has strong oxidation effect. Hydroxyl free radicals are cytotoxic and can kill tumor cells, which is the basis of chemical dynamic therapy. This process does not require additional external effects and is an effective treatment strategy.

The tumor microenvironment is characterized by weak acidity and high hydrogen peroxide concentration. However, the amount of hydrogen peroxide produced by hydroxyl radicals cannot achieve the sustained killing effect on tumors (Tang et al., 2019). Therefore, there are emerging researches on Fenton reaction catalysts or additional hydrogen peroxide (Lin et al., 2018; Sang et al., 2020). The study of nanozyme in promoting Fenton reaction is also a hot research topic, and they mainly focus on increasing the content of hydrogen peroxide and oxygen in the tumor microenvironment and reducing the overexpression of glutathione (Zhang et al., 2018; Fan et al., 2019), such as iron-based nanomaterials and MOF (Zhang et al., 2018). However, some processes are too complex to fully supplement hydrogen peroxide and oxygen, and the effect of the depletion of glutathione is not satisfactory.

Platinum nanozyme is one of the most studied nanozymes. Hao et al. (2020) developed a ROS-responsive prodrug nanoparticle (CPT-TK-HPPH/Pt NP) loaded with platinum nanozyme (PtNP). In this nanoplatform, PtNP is responsible for decomposing hydrogen peroxide to produce oxygen, alleviating hypoxia and bolstering PDT efficiency. While the prodrug is responsible the production of ROS which is a high effective for enhancing PDT and controlling the release behavior of CPT in the prodrug for the purpose of improving CDT.

The biomimetic CoO@AuPt nanozyme prepared by Fu et al. (2020) is stable under physiological conditions and can spontaneously disintegrate in the tumor microenvironment. It has tumor-specific targeting effect and less damage to normal cells. It can improve the ROS level in the tumor cells, decompose glucose to supply hydrogen peroxide, and consume local GSH. The effect of inhibiting tumor growth *in vivo* and *in vitro* experiment is remarkable.

Zhang et al. (2021) modified Au@Pt nanozyme onto bacterial surfaces, which could efficiently release ROS at tumor sites by exploiting the tumor-targeting properties of bacteria and the catalytic effect of Au@Pt nanozyme in a weak acidic environment. In addition, Au@Pt nanoenzyme produces ^1^O_2_ and O_2_^−^ in acidic TME which also enhances tumor CDT. Dying tumor cells can release tumor antigens and present them to T cells. At the same time, bacterial substrates increase the body’s immune stress and promote the production of T cells. Then T cells differentiate and mature into CD4^+^ or CD8^+^ T cells and secrete IFNγ, which can activate innate immune response and adaptive immunity. In conclusion, it provides a promising strategy to achieve highly accurate anticancer efficacy without significant toxic side effects.

### Photothermal therapy

Photothermal therapy (PTT) is a new non-invasive tumor treatment method (Melamed et al., 2015). After targeting tumor tissue, photothermal treatment converts light energy into heat energy causing damage to located tumors. However, when the temperature is too high, it will cause damage to the surrounding normal tissues as well. Thus, only lower temperatures can be put to use. Nanozyme materials have played a certain role in the low-temperature photothermal precise treatment. The CeO_2_/Au@Pt nanospheres were fabricated by packing CeO_2_ into core-shell Au@Pt and modifying the nanosphere surface with polyethylene glycol (PEG) (Tang et al., 2022). With catalytic-like and peroxidase-like dual enzymatic activities, CeO_2_ is able to relive hypoxia and anti-tumor by generating toxic hydroxyl radicals. Due to the special photothermal properties, Au@Pt is able to achieve targeted PTT. Thus CeO_2_/Au@Pt nanospheres can be used for photothermal catalytic synergistic treatment of tumors.

**FIGURE 5:**
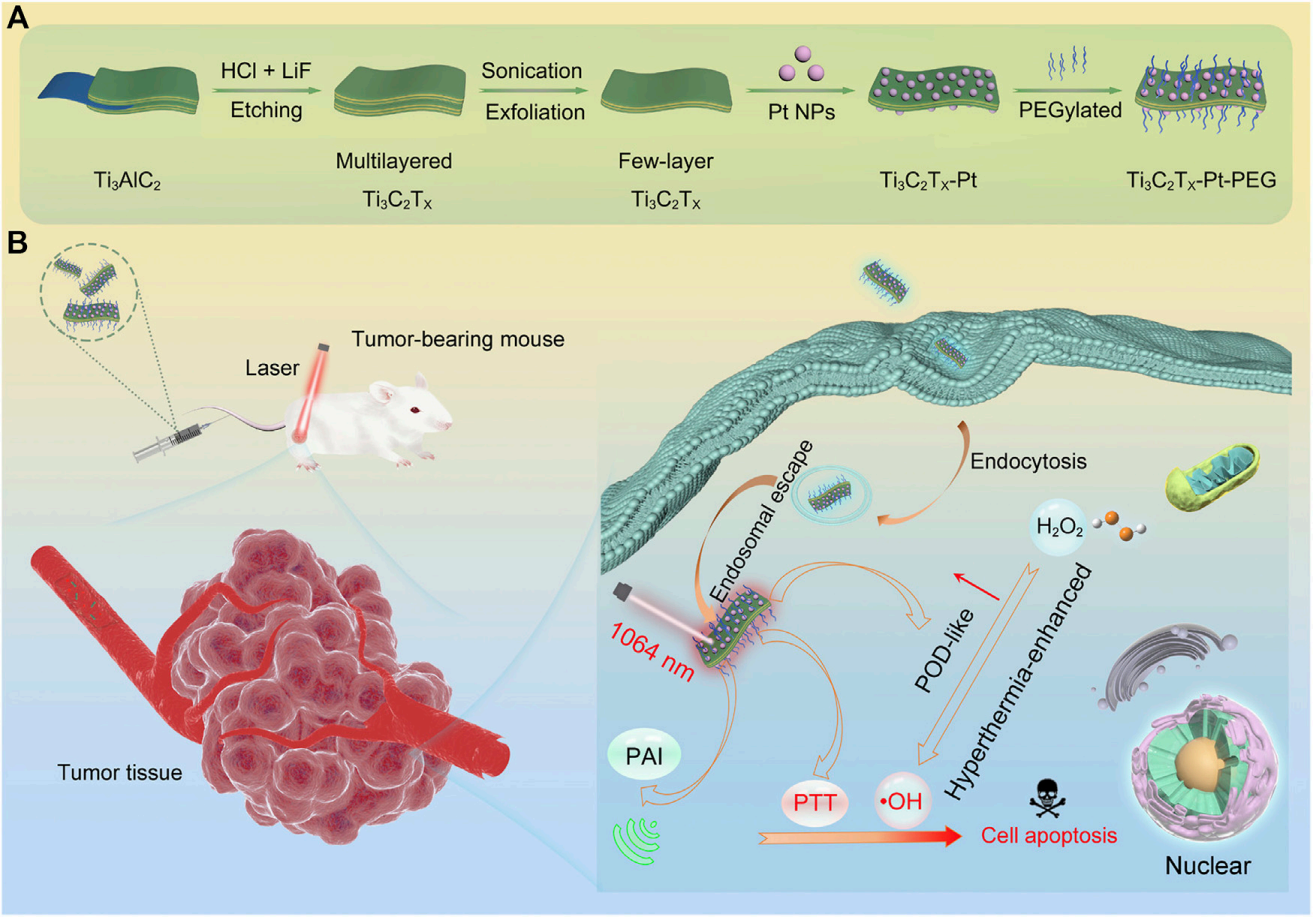
Design, fabrication, and catalytic-therapeutic schematic diagram of Ti3C2Tx-Pt-PEG. **(A)** Illustration of the synthetic procedure of Ti3C2Tx-Pt-PEG. **(B)** The schematic diagram of Ti3C2Tx-Pt-PEG with hyperthermia-enhanced nanozyme catalytic activity for cancer therapy (Zhu et al., 2022d). Copyright ^©^ 2022 American chemical society.

The Ti_3_C_2_T_x_-Pt-PEG composite nanomaterials prepared by Zhu et al., 2022d showed favourable photothermal properties when irradiated with NIR-II light at low power density (Figure 5). Moreover, the peroxidase-like activity of platinum nanoparticles was markedly enhanced by the temperature increase generated by the photothermal reaction of Ti_3_C_2_T_x_-Pt-PEG composite. This study offers a method for synergistic PTT/enzyme therapy, specifically on the basis of MXene nano-composite materials and metal catalysts.

**FIGURE 6:**
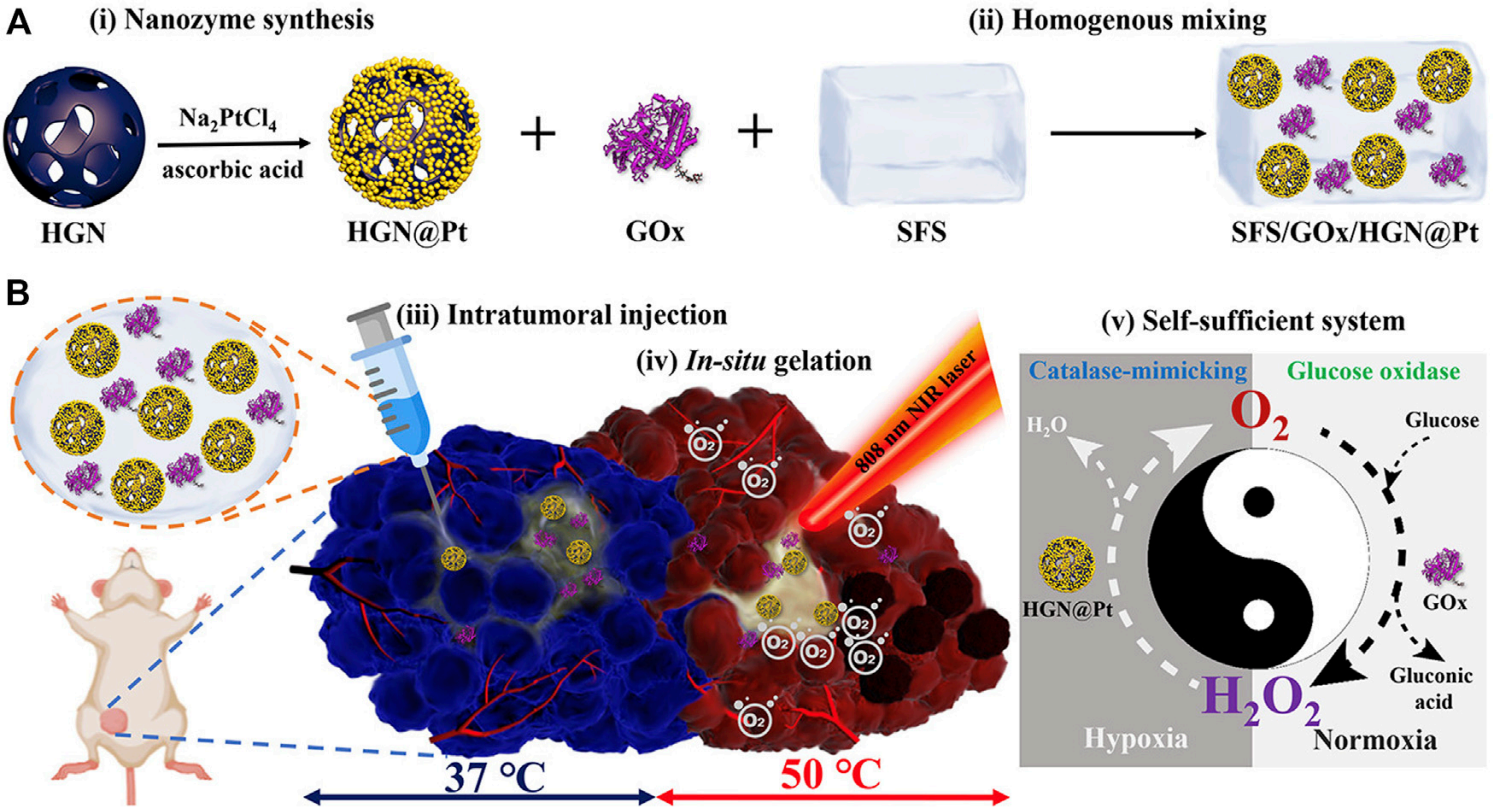
**(A)** Synthetic procedure and composition and **(B)** Schematic representation of the mechanism of the self-sufficient hybrid enzyme-based hydrogel system for the synergistic alleviation of tumor hypoxia and induction of the anti-cancer effect when combined with irradiation from an NIR laser (Wu et al., 2021). Copyright ^©^ 2021 American chemical society.

The silk fibroin hydrogel system based on mixed enzyme constructed by Wu et al. (2021) is a self-sufficient system. This system is composed of HGN@Pt nanocages and glucose oxidase (GO_X_) (Figure 6). HGN@Pt is much more efficient than Pt nanoparticles in catalyzing hydrogen peroxide under acidic conditions in the tumor microenvironment. In addition, HGN@Pt exhibits strong surface plasmon resonance peaks in the near-infrared wavelength range, so it is also a good photothermal agent in photothermal therapy. Besides, GO_X_ can consume glucose to improve starvation treatment.

### Starvation therapy

**FIGURE 7:**
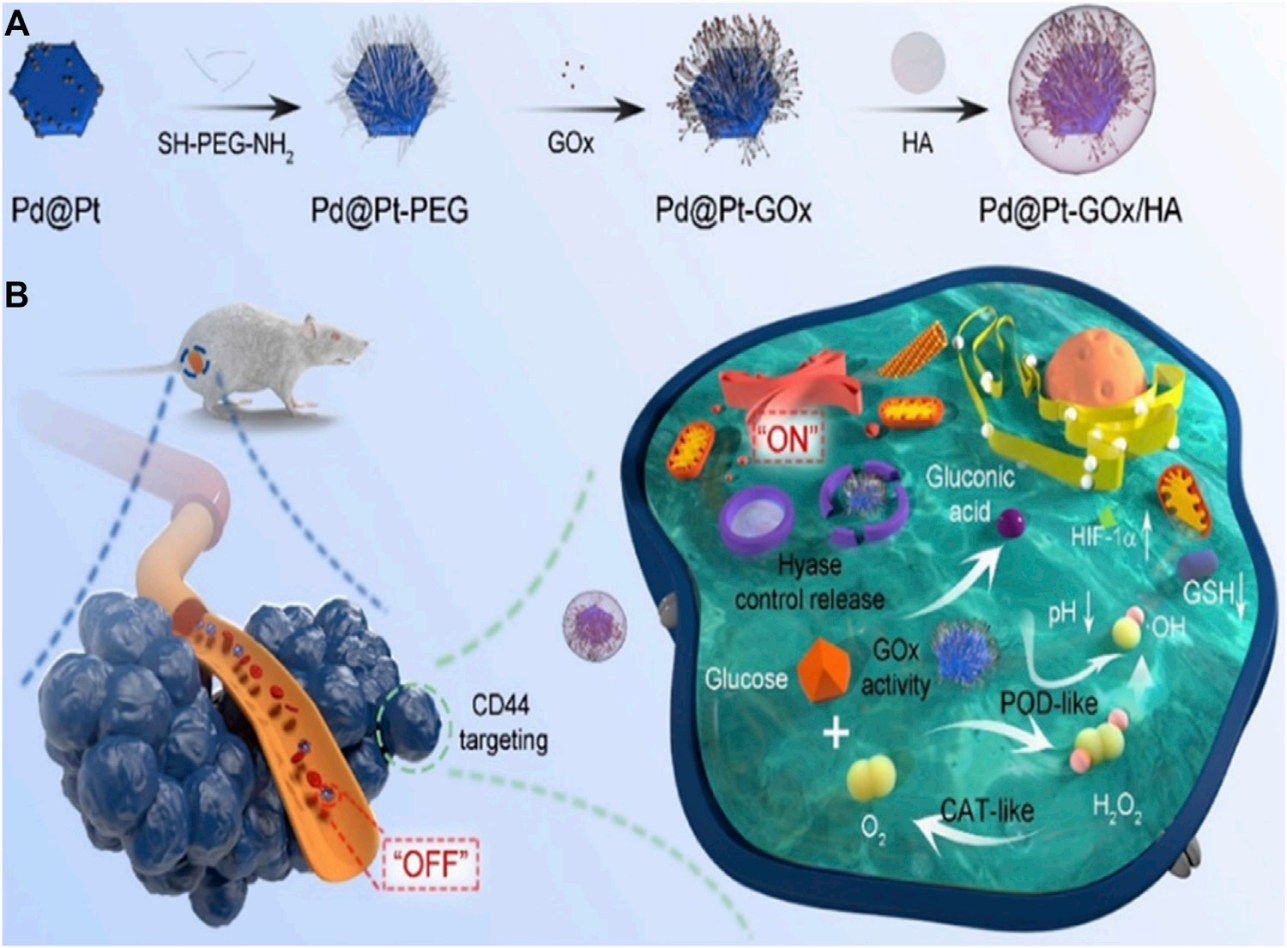
Schematic illustration of the synthesis procedure **(A)** and nanozyme-mediated starving-enhanced chemodynamic cancer therapy **(B)** of the Pd@Pt-GOx/HA Nanoreactors (Ming et al., 2020). Copyright ^©^ 2020 American chemical society.

Starvation therapy was first proposed by Professor Fokman of Harvard University in 1971. This theory believes that the occurrence and development of tumors are closely related to the adjacent nutrient vessels, so cutting off the blood vessels near the tumors can block the nutrient supply of tumor cells to a certain extent, which will lead to the death of tumors. The effect of starving and killing tumor cells can be achieved through certain methods, such as increasing glucose and oxygen consumption. GOx is the enzyme used to consume glucose and oxygen. However, this treatment might also affect and even kill normal cells. Pd@Pt-GOx/HA developed by Ming et al. (2020) is a product of the combination of composite nanomaterials with multiple nanozyme activities and glucose oxidase (Figure 7). It can not only produce glucose, oxygen, hydrogen peroxide and glucuronic acid by the action of glucose oxidase, but also produce hydroxyl by the action of catalase of composite nanozymes. Hydroxyl has a killing effect on tumor cells and inhibits tumor growth (Ming et al., 2020). GSH is also one of the important metabolites in tumor cells. By inhibiting the metabolism of GSH, tumor cells can also be affected. However, there are few studies on platinum nanozymes in this field (Zhu et al., 2022c).

In addition, there is also Au metal capable of enhancing the catalysis of glucose oxidase, but its catalytic activity is low because of the limited electrons distribution on the surface. However, AuPt nanozyme can transfer the electrons on Auto Pt (Chen and Song, 2022), so that its catalytic efficiency is far higher than that of Au nanozyme.

Starvation therapy can also kill tumor cells by increasing the activity of glucose oxidase. The multi-functional mimetic hybrid nanomaterial (ICG/Au/Pt@PDA−PEG) by Ciou et al. (2021) is synthesized from the nanoenzymes AuNPs (glucose oxidase activity), PtNPs (peroxidase and catalase activity) and the photosensitizer indocyanine green (ICG) immobilized on the surface of polydopamine (PDA) (ICG/Au/Pt@PDA—PEG) which can be used for starvation therapy, chemodynamic therapy and phototherapy. Experiments *in vivo* and *in vitro* have proved that this nanohybrid system has superior cooperative effect on killing tumor cells and causing less damage to peripheral normal tissues.

## Other applications

### Inhibition of tumor metastasis

In recent years, it was reported that Pt NPs can not only promote catalase catalysis and increase oxygen, but also inhibit the metastasis of tumor cells. For example, dendrimer-encapsulated Pt nanoparticles have been shown to suppress tumor metastasis, invasion and adhesion by affecting the mRNA level of EMT (An et al., 2021).

### Used to clinical diagnosis

A variety of nanomaterials combined with photothermal agents show excellent disease treatment and diagnosis effects. Pt nanomaterials are one of them. Platinum-doped Prussian blue (PtPB) nanozymes was obtained by a simple method of *in situ* reduction of PtCl_6_^2−^ on the prussian blue nanocubes (PB) surface (Li et al., 2021b). This PtPB nanozymes showed adjustable localized surface plasmon resonance (LSPR) frequencies that not only dramatically improve photothermal convertibility but also enable multi-wavelength photoacoustic/infrared thermography-guided photothermal therapy.

## Conclusion and opinions

Since the discovery and research of platinum nanozyme materials, hundreds of platinum nanozymes and platinum metal related synthetases have been successively invented and applied in the field of medicine, especially in tumor treatment. These applications mainly include radiotherapy, photodynamic therapy, sonodynamic therapy, chemical dynamic therapy, photothermal therapy, and starvation therapy, etc. However, the researches on platinum nanozyme materials still face certain challenges.

As an artificial nano biomimetic enzyme, platinum nanozyme has significant advantages:

1) Physically and chemically stability: Platinum nanozyme can withstand the acidic environment in the tumor microenvironment, and platinum-related synthetase can provide precise treatment in a lower temperature environment. Platinum nanozymes also have excellent photothermal properties, which can promote the death tumor cells when irradiated by radiation.
2) Platinum nanozymes targeting tumor cells can precisely aggregate in tumor cells, promote the decomposition of hydrogen peroxide in the tumor microenvironment, and increase ROS content.
3) Low cost: Platinum nanozyme, as an artificial biomimetic nanozyme, has superior advantages in manufacture, including simple preparation, low cost, easy access, etc.
4) Low toxicity *in vivo*: Compared with the side effects of tumor chemotherapy drugs such as immunosuppression, and the damage on normal tissue caused by radiotherapy, platinum nanozymes has fewer side effects on the immune system, and because of the higher targeting effect of platinum nanozymes, the damage to the surrounding normal tissue cells will also be reduced. Besides, Platinum nanozymes are small and easily metabolized by the body.
5) Improving the tumor microenvironment: Platinum nanozyme can promote the decomposition of endogenous hydrogen peroxide and generate oxygen, consequently alleviate the hypoxia state in the tumor microenvironment and improve the impact of acidic environment to a certain extent.

There are still a series of challenges in front of platinum nanozymes research.

The application of platinum nanozymes is limited. Compared with other metal nanozymes such as iron nanozymes, platinum nanozymes are mainly used in the field of tumor therapy, such as photodynamic therapy and radiotherapy sensitization. This could be a major obstacle to broadening the clinical usage of platinum nanozymes.

Collectively, the role of platinum nanozymes cannot be underestimated in the field of novel treatment for tumors. Further exploration is required to improve the targeting and therapeutic efficiency of platinum nanozymes, so as to exploit the new role and advantages within platinum nanozymes.
